# Molecular Biological Comparison of Pulp Stem Cells from Supernumerary Teeth, Permanent Teeth, and Deciduous Teeth for Endodontic Regeneration

**DOI:** 10.3390/ijms26051933

**Published:** 2025-02-24

**Authors:** Hui Lu, Fangyang Shi, Boqun Wang, Yexin Zheng, Jiaxuan Lu, Binghui Zeng, Wei Zhao

**Affiliations:** 1 Hospital of Stomatology, Guanghua School of Stomatology, Sun Yat-sen University, Guangzhou 510055, China; luhui7@mail2.sysu.edu.cn (H.L.); shify6@mail2.sysu.edu.cn (F.S.);; 2 Guangdong Provincial Key Laboratory of Stomatology, Guangzhou 510055, China

**Keywords:** biological phenotype, cell vitality, odontogenic differentiation, transcriptome, supernumerary tooth-derived pulp stem cells, DPSCs, SHED

## Abstract

Supernumerary tooth-derived pulp stem cells (SNTSCs) hold promise for endodontic regeneration, yet little is known about the similarities and diversities of SNTSCs relative to other dental-derived mesenchymal stem cells. Herein, we compare the biological characteristics of SNTSCs with dental pulp stem cells (DPSCs) and stem cells from human exfoliated deciduous teeth (SHED). Cell proliferation, migration, and odontogenic differentiation potential, as well as viability and aging-related phenotype after long-term storage, were evaluated. Additionally, gene expressions during induced odontogenic differentiation were profiled by transcriptome sequencing. Our findings indicated that the SNTSCs outperformed the DPSCs but were inferior to the SHED in cell proliferation. The SNTSCs exhibited comparable migratory capacity to the SHED and surpassed the DPSCs. Of particular interest, the odontogenic differentiation potential followed the pattern of SHED > SNTSCs > DPSCs. After two years of storage, the SNTSCs showed weakness in resistance to apoptosis induced by lipopolysaccharide, whereas difference between the SNTSCs and SHED in stemness and senescence was not obvious. Transcriptome analysis revealed that upregulated genes in the SNTSCs were particularly enriched in inflammatory signaling pathways compared to both the DPSCs and SHED. Collectively, SNTSCs share many satisfactory features in proliferation and differentiation with SHED, which may serve as a promising alternative cell source for endodontic regeneration.

## 1. Introduction

Dental caries, trauma, and developmental anomalies frequently result in pulp necrosis. Stem cell-based tissue engineering presents a novel approach for regenerative endodontic treatment. Dental-derived mesenchymal stem cells (DMSCs) have emerged as promising candidates for cell-based therapy owing to their easy accessibility and minimal invasiveness [1]. Human dental pulp stem cells, derived from the ectodermal lineage, are originated from migrating neural crest cells. They possess the capability to differentiate into odontoblasts and generate dentin-like tissue, making them important cellular sources for dentin-pulp regeneration. Typically, these cells include dental pulp stem cells isolated from extracted permanent teeth (DPSCs) [2] and stem cells from human exfoliated deciduous teeth (SHED) [3], which are considered heterogeneous populations [4].

Supernumerary teeth, distinct from conventional permanent teeth and deciduous teeth, are defined as any teeth formed in excess to the regular number of teeth in the dentition, with a reported incidence of 0.2~6.7% [5,6,7,8,9]. Mesiodens is the most common type of supernumerary tooth [10]. Due to the similarities between supernumerary teeth and odontomas concerning their topographic distribution, pathologic manifestations, and genetic factors [11], some researchers propose that an odontoma can be considered a type of supernumerary tooth [12]. A single supernumerary tooth may represent a developmental dental anomaly, while multiple supernumerary teeth are often genetically related [13]. Inactivation of adenomatous polyposis coli (APC) or abnormal activation of Wnt/β and Shh signaling are believed to play important roles in the formation of supernumerary teeth [14,15,16]. Given that the formation of supernumerary teeth is widely considered to be associated with hyperactivity of the dental lamina [17], it is meaningful to explore whether stem cells from supernumerary teeth possess superior potential for odontogenesis.

As a newly discovered type of DMSCs, supernumerary tooth-derived pulp stem cells (SNTSCs) have been identified as a population distinct from DPSCs since the initial report in 2008 [18]. SNTSCs not only share similar non-invasive sampling properties with SHED but also are more accessible due to the larger pulp tissue within supernumerary teeth compared to deciduous teeth [19]. This makes SNTSCs a potential cell source for tissue engineering and regenerative medicine. Although stem cells from the pulp, follicle, and apical papilla of supernumerary teeth have been characterized [20,21,22], comprehensive evaluation studies on SNTSCs are limited. Lee et al. evaluated the proliferation and migration of SNTSCs and SHED, showing a slight superiority of SHED in these two aspects [22]. A study by Lu et al. demonstrated that the proliferation capacity of SNTSCs was slightly stronger than that of DPSCs [23]. Apart from the basic characteristics such as migration and proliferation, the ability to differentiate into a mineralizing odontoblast-like phenotype is particularly important in the endodontic regenerative applications. However, the odontogenic differentiation potential of SNTSCs has not been fully elucidated. The question remains whether SNTSCs are equally suitable for endodontic regeneration as DPSCs and SHED. Much remains to be discovered about the genetic regulation during the differentiation of the three cell types.

In this study, we hypothesized that SNTSCs possess superior biological properties, especially the odontogenic differentiation potential, which may serve as promising seed cell source for endodontic regeneration. To confirm this hypothesis, SNTSCs, DPSCs, and SHED were isolated. A series of assays was performed to compare the proliferation, migration, and, particularly, the odontogenic differentiation of the three cell types. The apoptosis, stemness, and senescence of the cells were evaluated after two years of storage. Additionally, transcriptome analysis was employed to reveal the transcriptomic characteristics of these cells during the odontogenic differentiation. This study provides a comprehensive evaluation on the molecular biological characteristics of SNTSCs, DPSCs, and SHED, aiming to explore the possibility of SNTSCs as a cell candidate for endodontic regeneration. Insights into the molecular biological characteristics of these DMSCs will pave the way for optimizing stem cell-based therapy.

## 2. Results

### 2.1. Cell Morphology and Immunophenotype

**Figure 1:**
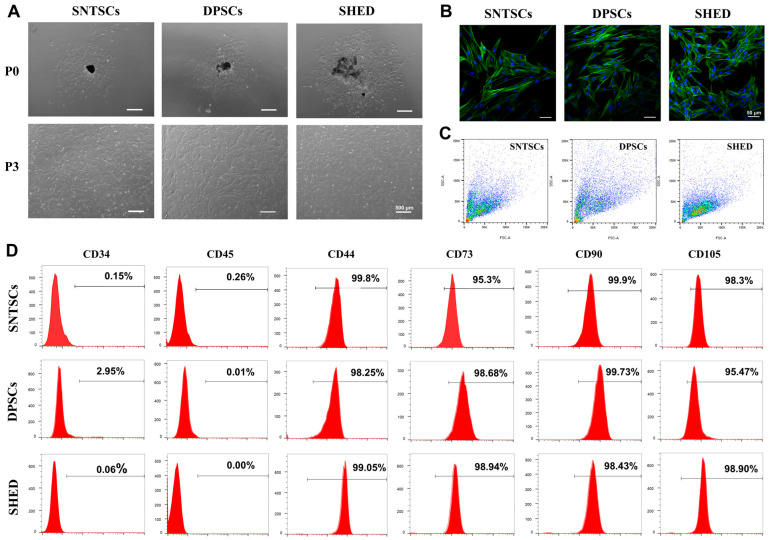
Characteristics of supernumerary tooth-derived pulp stem cells (SNTSCs), dental pulp stem cells (DPSCs), and stem cells from human exfoliated deciduous teeth (SHED). (**A**) Typical morphology of SNTSCs, DPSCs, and SHED (P0 and P3), bar = 500 μm. (**B**) The fluorescent images of SNTSCs, DPSCs, and SHED at P3. Cytoskeleton and nucleus were stained with phallotoxins (green) and DAPI (blue), respectively, bar = 50 μm. (**C**) Sizes of SNTSCs, DPSCs, and SHED at P3 analyzed by flow cytometry. (**D**) Expression of cell surface markers.

Stem cells were isolated from the pulp of supernumerary teeth (donor age: 5–7 years old), third molars (donor age: 20–30 years old), and human exfoliated deciduous teeth (donor age: 5–8 years old). The three cell types—SNTSCs, DPSCs, and SHED—showed indistinctive signatures in morphology, displaying a spindle-like or fibroblast-like appearance (Figure 1A,B). Flow cytometric analysis revealed that the SNTSCs and SHED were slightly smaller in size compared to the DPSCs (Figure 1C). All three cell types expressed homologous surface markers, including positive expression for MSC markers CD44, CD73, CD90, and CD105 and negative expression for CD34 and CD45 (Figure 1D).

### 2.2. Cell Growth and Clonogenic Population

**Figure 2:**
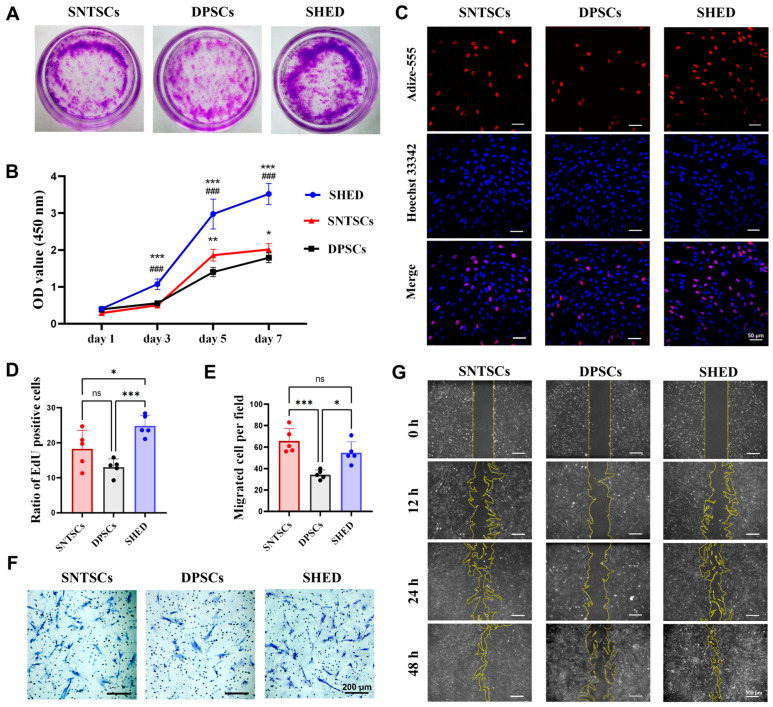
Proliferation and migration of SNTSCs, DPSCs, and SHED. (**A**) Colony-forming ability of SNTSCs, DPSCs, and SHED at P3. (**B**) Proliferation of SNTSCs, DPSCs, and SHED at P3 detected by CCK-8 assay (* *p* < 0.05, ** *p* < 0.01, and *** *p* < 0.001 vs. DPSCs; ### *p* < 0.001 vs. SNTSCs). (**C**) Representative images showing the proliferation abilities of SNTSCs, DPSCs, and SHED at P3 detected by EdU assay on day 3 (Scale bar = 50 μm). (**D**) Quantitative assessment of EdU-positive cells on day 3 detected by EdU assay (* *p* < 0.05 and *** *p* < 0.001). (**E**) Quantitative comparison of migrated cells per field in transwell assay (* *p* < 0.05 and *** *p* < 0.001). (**F**) Representative images showing the migration capacity of cells at P4 tested by the transwell assay (Scale bar = 200 μm). (**G**) Representative images of scratch wound healing monitored for 48 h (Scale bar = 500 μm). Data are presented as the mean ± standard deviation. ns, no significance.

The colony-forming unit (CFU) assays revealed significant differences in clonogenic ability among the SNTSCs, DPSCs, and SHED. Over a period of 10 days, the SHED formed more colony units than both the SNTSCs and DPSCs (Figure 2A). The Cell Counting Kit-8 (CCK-8) assays demonstrated that there were significant differences in proliferative capacity on day 3, 5, and 7 with SHED > SNTSCs > DPSCs (Figure 2B and Table S1). This similarity was further confirmed by EdU assays. On day 3, the proportion of EdU-positive cells was in the order of SHED > SNTSCs > DPSCs, with statistically significant differences observed between SHED and SNTSCs (24.80% vs. 18.28%, *p* < 0.05) and between SHED and DPSCs (24.80% vs. 13.04%, *p* < 0.001) (Figure 2C,D and Table S2).

### 2.3. Cell Migration

The transwell assay results indicated no significant difference in migration ability between the SNTSCs and SHED (Figure 2E,F). However, the migration ability of the DPSCs was significantly weaker than that of both the SNTSCs and SHED (34.4 ± 4.28 vs. 65.8 ± 11.52, *p* < 0.001 and 34.4 ± 4.28 vs. 54.8 ± 10.23, *p* < 0.05, respectively; Table S3). To avoid the influence of cell size on the results, a scratch assay was also performed. Microscopic images of wound healing showed continuous sheath migration over 48 h for the SNTSCs, DPSCs, and SHED. After 24 h, the migration rates of the SNTSCs and SHED were notably higher than those of the DPSCs (Figure 2G). These results suggest that SNTSCs exhibit a migration capacity comparable to that of SHED.

### 2.4. Odontogenic Differentiation Potential

**Figure 3:**
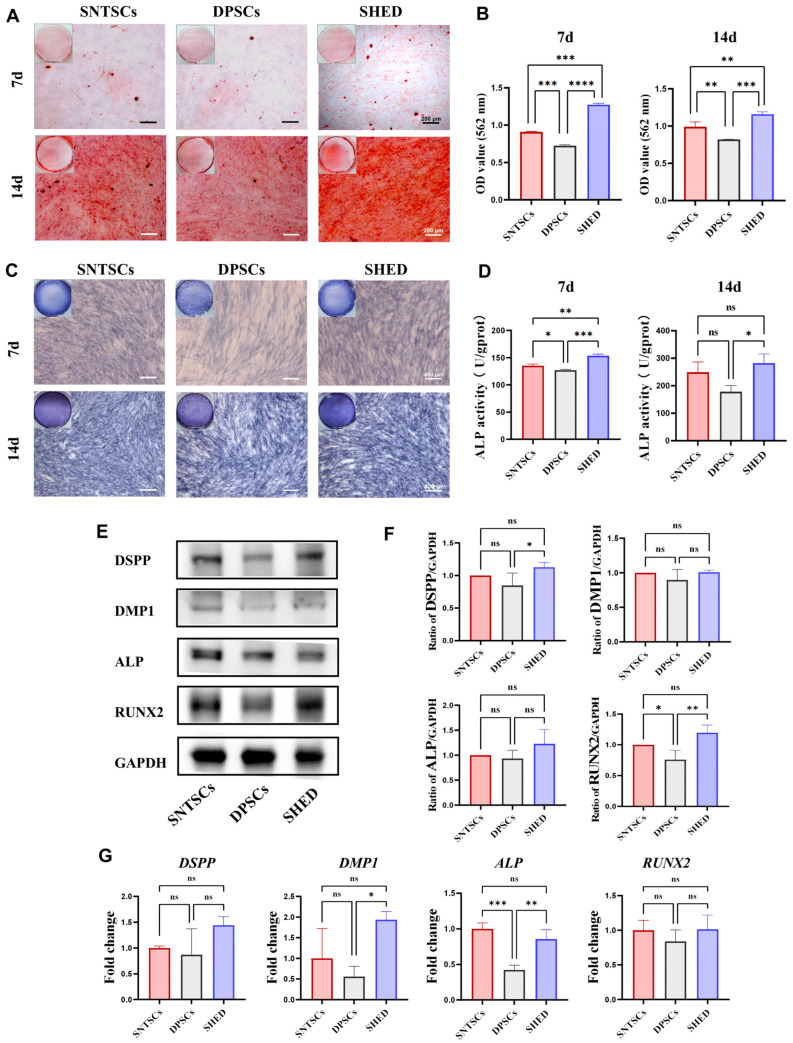
Assessment of odontogenic differentiation potential of SNTSCs, DPSCs, and SHED (at P5). (**A**) Gross appearance and microscopic images of Alizarin red staining for 7 and 14 days, scale bar = 200 μm. (**B**) Quantitative analysis of Alizarin red staining for 7 and 14 days. (**C**) Gross appearance and microscopic images of Alkaline phosphatase (ALP) staining for 7 and 14 days, scale bar = 400 μm. (**D**) Quantitative detection of ALP activity. (**E**) Expression levels of odontogenic-relative proteins for 14 days. (**F**) Quantification of the gray signal intensity based on the Western blot on day 14 (*n* = 4). (**G**) Expression levels of odontogenic genes tested by qRT-PCR on day 14. Data are presented as the mean ± standard deviation. * *p* < 0.05, ** *p* < 0.01, *** *p* < 0.001, and **** *p* < 0.0001. ns, no significance.

To investigate the odontogenic differentiation potential of the SNTSCs, DPSCs, and SHED, these cells were cultured with an odontogenic induction medium. Alizarin red staining, alkaline phosphatase (ALP) staining, and ALP activity assessment were conducted following 7 and 14 days of odontogenic induction. Early mineralized module formation was observed in the SNTSCs, DPSCs, and SHED using the Alizarin red staining at magnification (Figure 3A). The quantification of staining revealed that on both day 7 and day 14, the SHED exhibited a higher level of mineralization formation, followed by the SNTSCs and then the DPSCs (Figure 3B). The ALP staining indicated that the SNTSCs formed more uniform and denser mineralization nodules compared to the DPSCs (Figure 3C). A similar pattern of significantly greater ALP activity for SHED > SNTSCs > DPSCs was detected on day 7, whereas no significant difference in ALP activity between the SNTSCs and SHED was noted on day 14 (Figure 3D and Table S4).

The expression levels of odontogenic-related markers, including dentin siolophosphoprotein (DSPP), dentin matrix protein 1 (DMP1), ALP, and runt-related transcription factor 2 (RUNX2), were assessed. Western blot analysis showed that after 14 days of odontogenic induction, the protein expression levels of DSPP and RUNX2 were significantly higher in the SHED compared to the DPSCs. The expression of RUNX2 in the SNTSCs was slightly lower than that in the SHED (Figure 3E,F). The superiorities of the SHED and SNTSCs in odontogenic differentiation were also verified through qRT-PCR. In contrast to the DPSCs, *ALP* expression was notably upregulated in the SNTSCs, while both *DMP1* and *ALP* expression were significantly increased in the SHED. However, there was no significant difference in the expression of *DSPP*, *DMP1*, *RUNX2*, and *ALP* between the SNTSCs and the SHED (Figure 3G).

### 2.5. Cell Vitality After Two Years of Storage

**Figure 4:**
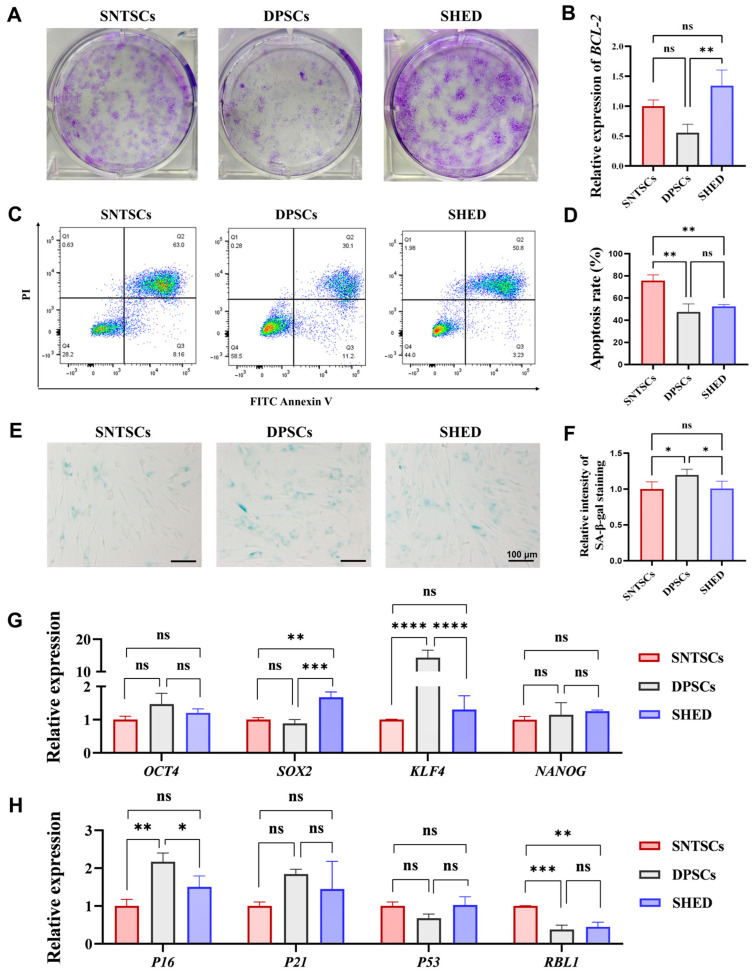
The apoptosis, stemness, and senescence of cells after storage for two years. (**A**) Representative images of colony-forming unit for SNTSCs, DPSCs, and SHED at P5 after two years’ freezing. (**B**) mRNA expression of anti-apoptosis-related gene *BCL-2* in cells at P5. (**C**) Cell apoptosis was determined using Annexin V-FITC/PI double staining after incubation with 10 μg/mL lipopolysaccharide (LPS) for 24 h. (**D**) Apoptosis rates of SNTSCs, DPSCs, and SHED, including early apoptosis (Annexin-V+ PI−) and late apoptosis (Annexin-V+ PI+). (**E**) Senescence-associated β-galactosidase (SA-β-gal) staining of cells at P10 (Scale bar = 100 μm). (**F**) Semi-quantitative analysis of SA-β-gal staining. (**G**) mRNA expression of stemness-related genes in cells at P10. (**H**) mRNA expression of senescence-related genes in cells at P10. Data are presented as the mean ± standard deviation. * *p* < 0.05, ** *p* < 0.01, *** *p* < 0.001, and **** *p* < 0.0001. ns, no significance.

Cell vitality of the SNTSCs, DPSCs, and SHED after two years of cryopreservation was assessed via CFU assay and apoptosis assay. The clonogenic abilities of all the cell types exhibited varying degrees of decline following storage, with a significant reduction observed in the DPSCs (Figure 4A). Additionally, the expression level of the anti-apoptosis gene *BCL-2* was significantly higher in the SHED compared to both the SNTSCs and DPSCs, while no significant difference was found between the SNTSCs and DPSCs (Figure 4B). Lipopolysaccharide (LPS)-induced apoptosis was detected via flow cytometry. Under inflammatory stimulation, the SNTSCs demonstrated the highest level of cell apoptosis (FITC-Annexin V+), while the DPSCs exhibited the lowest rate of late apoptosis (FITC-Annexin V+ PI+) (Figure 4C,D and Table S5).

### 2.6. Cell Senescence and Stemness After Two Years of Storage

Cell senescence at passage 10 (P10) was evaluated through senescence-associated β-galactosidase (SA-β-gal) staining and expression of senescence-related genes. The results showed that senescent cells were more evident in the DPSCs post-storage, which were stained with blue (Figure 4E). Quantitative analysis revealed that the levels of SA-β-gal in the DPSCs were slightly higher than those in the SNTSCs and SHED (Figure 4F). Furthermore, mRNA analysis of senescence-related genes showed that *P16* expression was higher in the DPSCs compared to both the SNTSCs and SHED, while *RBL1* expression was elevated in the SNTSCs (Figure 4H).

To assess cell stemness at P10 after cryopreservation, the expression of stemness genes (including *OCT4*, *SOX2*, *KLF4*, and *NANOG*) was examined (Figure 4G). The results showed that *SOX2* expression was significantly higher in the SHED compared to the SNTSCs and DPSCs (*p* < 0.01 and *p* < 0.001), whereas *KLF4* expression was significantly higher in the DPSCs compared to the SNTSCs and SHED (*p* < 0.0001). These findings indicate that DPSCs exhibit relatively high levels of cell senescence and weaker stemness.

### 2.7. Transcriptomic Characteristics

**Figure 5:**
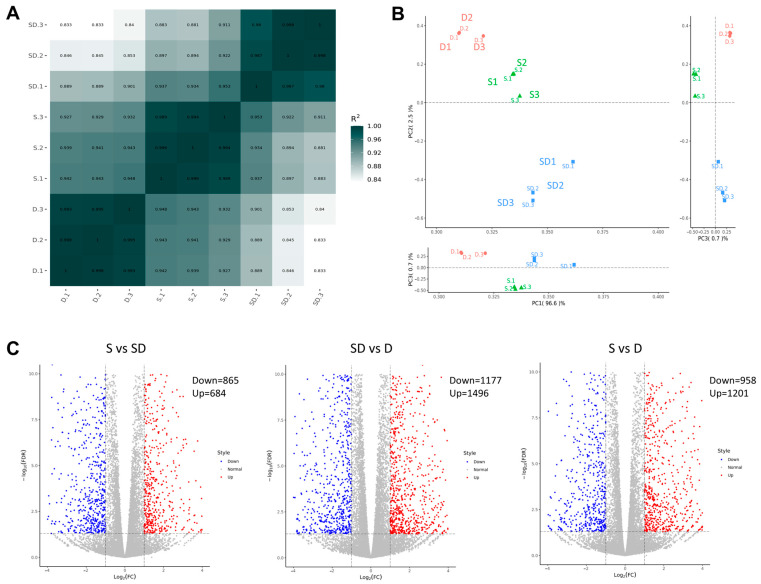
Comparison of gene expression profiling among SNTSCs, DPSCs, and SHED (at P5). (**A**) Correlation analysis of SNTSCs, SHED, and DPSCs by HeatMap diagram. (**B**) Principal component analysis (PCA) of SNTSCs, SHED, and DPSCs. (**C**) The distributions of gene expression in SNTSCs, SHED, and DPSCs based on |log2FC ≥ 1, *p*-value < 0.05. (SD: SNTSCs, D: DPSCs, S: SHED, *n* = 3).

To elucidate the variations among SNTSCs, DPSCs, and SHED during odontogenic differentiation, RNA sequencing and bioinformatic analyses were employed. Among these three cell types, the DPSCs and SNTSCs exhibited more diversity in gene expression profiling, which was confirmed by HeatMap and principal component analyses (PCA) (Figure 5A,B). Volcano plots indicated that the expression profile of the SNTSCs was more similar to that of the SHED compared to the DPSCs. There were 684 upregulated and 865 downregulated differential expression genes (DEGs) (|log2FC| ≥ 1, *p*-value < 0.05) in the SHED relative to the SNTSCs and 1496 upregulated and 1177 downregulated DEGs (|log2FC| ≥ 1, *p*-value < 0.05) in the SNTSCs compared to the DPSCs (Figure 5C). Taken together, these data reveal the variation in gene expression pattern among the three cell types, particularly between the SNTSCs and DPSCs.

### 2.8. Diversity of Signaling Pathways Enriched by DEGs

**Figure 6:**
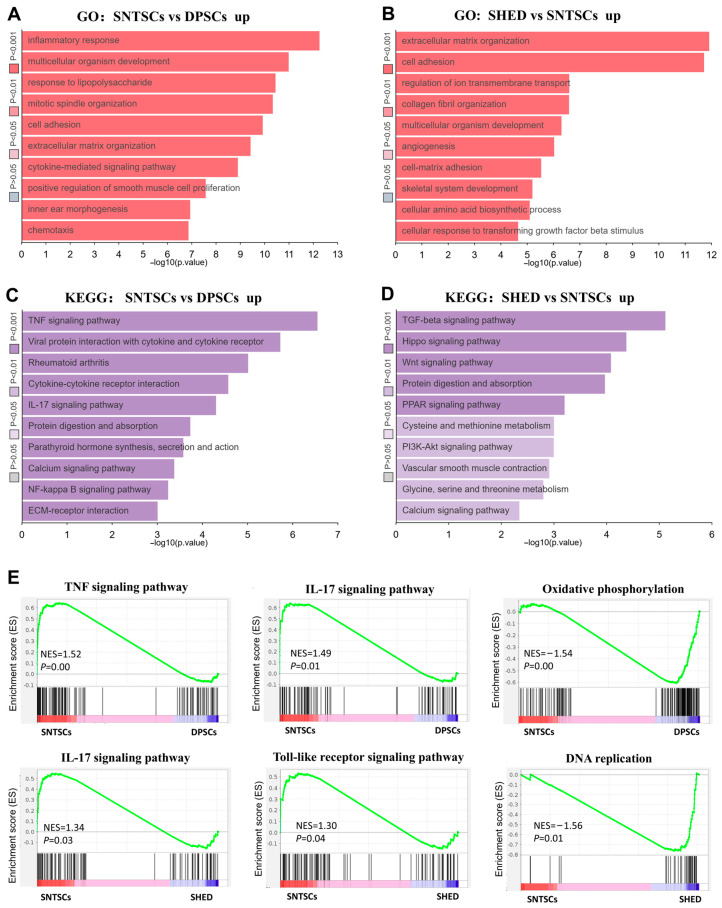
DEGs-associated gene ontology and signaling pathways. (**A**) Gene ontology (GO) analysis of upregulated genes in SNTSCs compared with DPSCs on the basis of biological processes. (**B**) GO analysis of upregulated genes in SHED compared with SNTSCs on the basis of biological processes. (**C**) Kyoto Encyclopedia of Genes and Genomes (KEGG) analysis of upregulated genes in SNTSCs compared with DPSCs. (**D**) KEGG analysis of upregulated genes in SNTSCs compared with DPSCs. (**E**) Genes enriched in representative pathways according to GSEA.

The differential expression analysis between the SNTSCs and DPSCs revealed that the upregulated DEGs in the SNTSCs were predominantly enriched in the biological process such as inflammatory response, multicellular organism development, response to lipopolysaccharide, mitotic spindle organization, and cell adhesion (Figure 6A). These upregulated DEGs were involved in the TNF signaling pathway, cytokine–cytokine receptor interaction, and IL-17 signaling pathway (Figure 6C). Meanwhile, the comparison between the SNTSCs and SHED highlighted a significant downregulated DEG in the SNTSCs associated with extracellular matrix functions, such as extracellular matrix organization, cell adhesion, collogen fibril organization, and angiogenesis (Figure 6B). The pathways enriched by these DEGs between the SNTSCs and SHED were enriched in those regulating proliferation and differentiation, such as TGF-β, Hippo, and Wnt signaling pathways (Figure 6D). Furthermore, gene set enrichment analysis (GSEA) demonstrated a strong association of TNF signaling (NES = 1.518, *p* = 0.0016) and IL-17 signaling (NES = 1.485, *p* = 0.0064) with the SNTSCs compared to the DPSCs. Particularly, the distinct distribution of DNA replication (NES = −1.558, *p* = 0.0054), IL-17 signaling (NES = 1.338, *p* = 0.0269), and Toll-like receptor signaling pathway (NES = 1.299, *p* = 0.0401) were revealed between the SNTSCs and SHED (Figure 6E). These results suggest that the SNTSCs were more sensitive to inflammatory stimuli and that the SHED exerted a higher level of biogenic activity at the transcriptional level.

## 3. Discussion

MSCs derived from diverse dental pulp origins might exhibit distinct biological characteristics, thereby influencing their preferred clinical applications [4]. Owing to the relatively low incidence of supernumerary teeth, research into the biological properties of SNTSCs remains limited. The potential of SNTSCs as novel seed cell sources for regenerative therapies is yet to be elucidated. This study characterized the proliferation, migration, stemness, and in vitro odontogenic differentiation ability of SNTSCs, compared them with DPSCs and SHED, and further dissected the similarities and diversities based on transcriptomic analyses. These findings offer cellular and molecular insights into SNTSCs as alternative MSC sources for regenerative endodontic therapy.

The capacity for stable self-renewal in stem cells underpins their differentiation potential. It is well known that SHED and DPSCs exhibited high proliferation rates, approximately 50% and 30% higher, respectively, compared to bone marrow mesenchymal stem cells (BMMSCs) [24]. In this study, SNTSCs outperformed DPSCs in terms of cell proliferation and CFU efficiency but were inferior to SHED, which is consistent with previous findings [22,23]. Additionally, cell location is one of the factors affecting their proliferative ability. It has been reported that there are more CD105-positive cells in the root of mesiodents than in the crown, which possessed higher CFU and proliferation capabilities [10]. The variations in cell growth may relate to differing expression levels of the mediators involved in the cell cycle progression from G1 to S phase [25]. According to GSEA, DNA replication is more closely associated with SHED than with SNTSCs, suggesting higher proliferative activity in SHED.

Another crucial aspect for the clinical application value of MSCs is their differentiation potential [26]. Previous studies have shown that compared to DPSCs, SHED express higher levels of odontogenic and osteogenic markers, such as ALP, collagen type I (Col I), and osteocalcin (OCN) [27]. However, the odontogenic differentiation ability of SNTSCs remains underexplored. This study observed significant differences in Alizarin red staining and ALP staining on day 7 and 14, with SHED > SNTSCs > DPSCs, consistent with previous findings [22,28]. Different levels of odontogenic markers were detected during the odontogenic induction process. As expected, the SHED expressed higher levels of DSPP, DMP1, ALP, and RUNX2 compared to the DPSCs and SNTSCs. Conversely, Sabbagh J et al. found that DPSCs, derived from premolars, exhibited a phenotype closer to odontoblasts than SHED and expressed higher levels of osteogenic and odontogenic differentiation markers [29]. Such discrepancies in odontogenic differentiation capacity across studies may be attributed to variations in anatomical sources. Biological variations among DMSCs related to anatomical localizations might thus exist.

Maintaining the stemness of MSCs after long-term cryopreservation, particularly their vitality and proliferation, is important for their clinical utility. Therefore, we evaluated CFU levels and cell apoptosis after two years of storage in this study. Among the three cell types, the SHED displayed the highest CFU efficiency following two years of cryopreservation, indicating that SHED possesses satisfactory biological stability [30]. The results are similar to those observed by Lee et al. [22]. Lee et al. compared the growth rates of SNTSCs and SHED post two years of storage and found the growth rate of the SNTSCs decreased, while that of the SHED remained nearly unchanged, suggesting that SNTSCs are inferior to SHED for long-term banking. Regarding cell apoptosis, our study demonstrated that the SNTSCs showed weakness in anti-apoptotic ability under LPS stimulation. The high sensitivity of the SNTSCs to inflammatory stimuli was further corroborated by gene ontology (GO) analysis and Kyoto Encyclopedia of Genes and Genomes (KEGG) analyses. Considering that the SNTSCs showed disadvantages over the SHED in long-tern storage and resistance to LPS-induced apoptosis, caution should be exercised in the future clinical applications of SNTSCs.

Aside from the differences observed in CFU ability and apoptosis, this study also focused on the expression of stemness genes and senescence-related phenotype after two years of cryopreservation. SOX2, OCT4, and NANOG are well-known stemness-related genes crucial for maintaining the pluripotency and self-renewal of MSCs [31,32]. Krüppel-like transcription factor 4 (KLF4) is a zinc-finger transcription factor involved in regulating proliferation and differentiation. It is highly expressed in non-dividing cells and associated with cell cycle arrest [33]. In this study, the mRNA expression level of KLF4 was notably higher in the DPSCs than in the SNTSCs and SHED, suggesting that cell division may be more inhibited in DPSCs. Among the senescence-related markers, Retinoblastoma-like 1 (RBL1) and P16 differed the most between the SNTSCs and DPSCs. RBL1 is considered a G1/S gene that is low in quiescent cells and high during the G1-S phase [34]. In this study, the DPSCs exhibited a higher level of P16 and lower level of RBL1, while there was no significant difference in the expression of senescence-related genes between the SNTSCs and SHED. This indicates that among the three types of cells, DPSCs may show a certain degree of cell cycle arrest, while SNTSCs maintain a relatively satisfactory performance state similar to SHED. In addition, although a statistical difference in SA-β-gal was revealed between the DPSCs and both the SNTSCs and SHED, this result might be meaningless from a biological perspective, as no signs of senescence for DPSCs were found in Barone’s study [35]. The disparities in senescence among the three cell types after cryopreservation remain to be further confirmed.

From the perspective of transcriptomes, this study delineated the similarities and diversities in gene expression profiling and signaling pathway among SNTSCs, DPSCs, and SHED. SNTSCs and SHED share many features in biological properties and follow a similar pattern of gene expression. Nonetheless, there are still differences between the two cell types in proliferation and odontogenic differentiation. As indicated by GSEA, compared to the SNTSCs, differentially expressed genes in the SHED were more enriched in DNA replication and extracellular matrix formation, explaining why SHED has higher proliferation and differentiation capabilities. On the other hand, compared to the DPSCs, the SNTSCs exhibited higher proliferative activity and migration capacity but had weaker anti-apoptotic abilities. Bioinformatics analysis indicated that the upregulated DEGs in the SNTSCs over the DPSCs were enriched in inflammatory response-related pathway such as TNF and IL-17 signaling pathway, suggesting that SNTSCs were more sensitive to inflammatory stimuli. This may explain why they exhibited the highest apoptosis rate in the apoptosis assay. Additionally, Lertruangpanya K et al. compared the protein profiles of dental pulp from supernumerary and normal permanent teeth, finding that the different functions of proteins were primarily related to cell apoptosis, cell death, healing, and vasculature development [36]. This indicates that cell viability, anti-apoptotic ability, and repair ability may be the main cellular biological differences between SNTSCs and DPSCs. This analysis will help elucidate the potential applications of DMSCs in tissue regeneration engineering.

This study has several limitations. First, due to the differences in the ages of donors from which the SNTSCs, DPSCs, and SHED were derived, this study could not rule out the potential impact of age and subject variation on the biological characteristics of stem cells. However, Lee et al. found that even SNTSCs and SHED obtained from the same individual exhibited differences in vitality and proliferation [22], suggesting that biological variations of DMSCs related to anatomical localizations might exist. Additionally, although the specific response of SNTSCs to inflammatory stimuli was identified through apoptosis assay and transcriptomic analysis, this study did not delve into the immunomodulatory capacity of SNTSCs. Despite this, relevant results from previous published articles can provide valuable insights. Conditioned media of DPSCs have demonstrated the ability to recruit monocytes and promote M2 polarization of macrophages, thereby playing a positive role in promoting angiogenesis [35]. Makino Y et al. [37] found that SNTSCs possess immunomodulatory capability in suppressing T cell viability and differentiation of Th17 cells, suggesting that SNTSCs are a promising MSC source for treating immune-related diseases. Furthermore, apoptotic extracellular vesicles (ApoEVs) released by SNTSCs were recently found to promote angiogenesis by transferring COL1A1, making SNTSC-ApoEVs a promising strategy for the treatment of angiogenesis-related diseases [38]. Future studies on the secretome and immunomodulatory properties of SNTSCs may further enhance their clinical utility.

## 4. Materials and Methods

### 4.1. Source of SNTSCs, DPSCs, and SHED

Supernumerary teeth, impacted third molars, and deciduous teeth were collected at the Department of Oral and Maxillofacial Surgery and the Department of Pediatric Dentistry in the Guanghua Hospital of Stomatology, Sun Yat-sen University. Informed consent was obtained from all subjects involved in the study, with approval from the Ethics Committee of the Affiliated Hospital of Stomatology, Sun Yat-sen University, Guangzhou, Guangdong, China. The collected teeth were free from caries and other pathological alterations. Subjects with the presence of systemic genetic diseases were excluded.

### 4.2. Cultivation of SNTSCs, DPSCs, and SHED

The extracted teeth were placed in pre-cooled phosphate-buffered saline (PBS; Biosharp, Nanjing, China) containing 1% penicillin/streptomycin (P/S; Gibco, Grand Island, NE, USA). Stem cells from the dental pulp of the teeth were isolated as previously described [39]. The cells were cultured in DMEM/F12 medium (Gibco) supplemented with 10% fetal bovine serum (FBS; Gibco) and 1% P/S at 37 °C. Cells at passages 3–5 (P3–P5) were used for experiments, ensuring consistency by using the same passage for the SNTSCs, DPSCs, and SHED.

### 4.3. Characterization of SNTSCs, DPSCs, and SHED

Cell morphology of the SNTSCs, DPSCs, and SHED at P0 and P3 was observed using an inverted microscope (Zeiss Axio Observer, Oberkochen, Germany). For better visualization, the cells were stained with phalloidin (Solarbio, Beijing, China) for cytoskeleton and 4,6-diamidino-2-phenylindole (DAPI; Beyotime, Shanghai, China) for nucleus. Fluorescent images of the SNTSCs, DPSCs, and SHED were captured with a confocal microscope (Zeiss LSM 780, Oberkochen, Germany).

Flow cytometry was employed to identify cell phenotypic markers. Cells at P3 (10^6^ cells/mL) were incubated with antibodies specific for CD34, CD44, CD45, CD73, CD90, and CD105 (Biolegend, San Diego, CA, USA) for one hour at 4 °C. Expression profiles were subsequently analyzed by flow cytometry (Beckman Coulter, Brea, CA, USA).

### 4.4. Cell Proliferation Assays

To compare the proliferative abilities of the SNTSCs, DPSCs, and SHED, a CFU assay, CCK-8 assay, and EdU proliferation kit were applied. For the CFU assay, cells at P3 were seeded at a concentration of 500 cells/mL in 6-well plates and cultured for 10 days. After being fixed with 4% paraformaldehyde and stained with 0.5% crystal violet, the colonies were recorded by photographs.

For the CCK-8 assay, cells at P3 were seeded at a density of 5000 cells per well in 96-well plates. After 1, 3, 5, and 7 days, cell proliferation was determined using the CCK-8 reagent (Dojindo, Tokyo, Japan) according to the manufacturer’s protocol. Optical density (OD) value at 450 nm was measured with a microplate reader (Biotek Epoch2, Winooski, VT, USA). The mean OD value of each group was averaged from three parallel wells. Three technical replicates were performed.

The EdU assay was conducted on SNTSCs, DPSCs, and SHED at P3 cultured on confocal dishes at 5000 cells per well. After 3 days, cell proliferation was labeled with an EdU proliferation kit (Beyotime) according to the manufacturer’s instructions. After the cell nuclei were stained with Hoechst33342, five random fields from each group were taken via laser confocal microscopy (Zeiss LSM 780, Germany). The proportion of EdU-positive nuclei in each group was calculated with ImageJ (version 1.50, National Institutes of Health, Bethesda, MD, USA).

### 4.5. Assessment of Migration Ability

To evaluate the migration ability, a transwell assay and scratch assay were performed. For the transwell assay, a suspension containing 2 × 10^4^ cells in 100 μL FBS-free α-MEM was added in the upper transwell chamber (pore size: 8 μm, Corning, NY, USA), while the lower chamber harbored 600 μL of α-MEM supplemented with 10% FBS. Migrated cells adherent to the lower membrane surface were appropriately fixed and stained with 0.1% crystal violet, and five random fields were later recorded with an inverted microscope.

For the scratch assay, cells at P4 were seeded into 6-well plates at a density of 2 × 10^5^ cells/well. After 24 h, an artificial scratch was created across the cell monolayers with 200-μL pipette tips. Afterwards, the medium was replaced with FBS-free medium to avoid the influence of cell proliferation. The closure of the induced gaps due to cell migration was monitored and recorded with an inverted microscope after 12, 24, and 48 h.

### 4.6. Alizarin Red Staining

To screen for the odontogenic differentiation potential of the SNTSCs, DPSCs, and SHED, cells at P5 were seeded into 6-well plates at a density of 2 × 10^5^ cells/well. When approximately 80% confluence was reached, the cells were treated with odontogenic induction medium (OIM) composed of α-MEM, 5% FBS, 1% P/S, 50 μM ascorbic acid (Sigma-Aldrich, St. Louis, MO, USA), 10 mM β-glycerophosphate (Sigma), and 0.1 μM dexamethasone (Sigma). After 7 days and 14 days, the cells were fixed with 4% polyformaldehyde and stained with Alizarin red solution (Cyagen Biosciences Inc., Guangzhou, China). The calcium nodules formed in each group were observed under an inverted microscope. For semi-quantitation, 10% cetylpyridinium chloride (Sigma) was added to each well and incubated for 30 min, followed by measuring the OD value at 562 nm.

### 4.7. Alkaline Phosphatase (ALP) Activity Assay and ALP Staining

Alkaline phosphatase (ALP) staining was also applied to assess the odontogenic differentiation potential of the three cell types. SNTSCs, DPSCs, and SHED at P5 were seeded in 12-well plates and treated with OIM for 7 and 14 days. A BCIP/NBT staining kit (Beyotime) was used according to the manufacturer’s protocol.

For ALP activity determination, SNTSCs, DPSCs, and SHED were seeded in 12-well plates and treated with OIM. The ALP activity was evaluated using an ALP assay kit (Nanjing Jiancheng Bioengineering Institute, Nanjing, China) on day 7 and 14 according to the manufacturer’s protocol.

### 4.8. Western Blot Analysis

Western blot analysis was performed to detect the protein expression levels of DSPP, DMP1, ALP, and RUNX2. SNTSCs, DPSCs, and SHED were cultured in OIM as described above for 14 days. Cells were then collected and lysed in RIPA buffer (KeyGen BioTECH, Nanjing, China) containing 1% protease inhibitor cocktail (CWBIO, Guangzhou, China). After determining protein concentrations using the BCA assay kit (CWBIO), proteins from each group were separated by 10% SDS-PAGE (GenSpirt, Nanjing, China) and transferred to polyvinylidene fluoride (PVDF) membranes (Millipore, Billerica, MA, USA). The membranes were blocked with 5% fat-free milk and subsequently incubated overnight at 4 °C with primary antibodies—anti-DSPP (1:500, Novus, CO, USA), anti-DMP1 (1:500, Bioss, Beijing, China), anti-RUNX2 (1:1000, Novus), anti-ALP (1:1000, Novus), and anti-GAPDH (1:1500, Novus)—followed by incubation with secondary antibody (1:5000, GNI, Tokyo, Japan) for one hour at room temperature. Each assay was repeated at least three times. The enhanced chemiluminescent (ECL) detection system (Millipore) was used, and ImageJ software was employed for quantitative analysis of protein expression.

### 4.9. Quantitative Real-Time PCR (qRT-PCR)

The expression levels of anti-apoptosis gene (*BCL-2*), stemness genes (e.g., *OCT4*, *SOX2*, *KLF4*, and *NANOG*), senescence-related genes (e.g., *P16*, *P21*, *P53*, and *RBL1*), and osteogenic genes (e.g., *DSPP*, *DMP1*, *ALP*, and *RUNX2*) were detected via qRT-PCR. Total RNA of the SNTSCs, DPSCs, and SHED was extracted using an RNA-Quick Purification Kit (YiShan Biotech, Guangzhou, China). cDNA synthesis was performed using a PrimeScript™ RT Master Mix (Takara Bio Inc., Kumamoto, Japan). qRT-PCR was conducted on a Light Cycler 96 Detection System (Roche, Basel, Switzerland) using the SYBR Green kit (Yeasen, Shanghai, China) according to the manufacturer’s protocols. GAPDH served as the control for normalizing RNA expression levels. The primer sequences of the indicated genes are available in Table 1.

### 4.10. Cell Apoptosis Assay

The apoptosis rates of the SNTSCs, DPSCs, and SHED after storage in 10% DMSO supplemented with FBS for two years were determined using an Annexin V-FITC and PI apoptosis detection kits (Dojindo, Tokyo, Japan). Cells at P5 were seeded in a 6-well plate at a density of 2 × 10^5^ cells per well. When approximately 80% confluence was reached, the cells were treated with 10 μg/mL LPS for 24 h to induce apoptosis. Afterwards, the cells were harvested, washed, and resuspended in 100 μL of binding buffer. Then, 5 μL FITC-Annexin V and 5 μL propidium iodide (PI) were added. Cells were incubated in the dark for 15 min. Flow cytometry (BD LSRFortessa, San Diego, CA, USA) was used to detect cell apoptosis. Data analysis was performed using FlowJo software (version 10.6, BD, NJ, USA).

### 4.11. SA-β-Gal Staining

Cells at P10 were seeded into 6-well plates with 10^5^ cells per well for 3 days. The SA-β-gal activity of the SNTSCs, DPSCs, and SHED was examined according to the manufacturer’s protocol (Beyotime). After being fixed with 4% PFA, the cells were incubated with SA-β-Gal staining solution at 37 °C for 24 h. The senescent cells were stained blue. Five random fields were captured by an inverted microscope and calculated using ImageJ.

### 4.12. RNA Sequencing and Bioinformatic Analyses

To further understand the different molecular events during odontogenesis, transcriptome-wide gene expression was profiled by transcriptome sequencing to dissect the similarities and diversities. Total RNA (*n* = 3) was extracted from SNTSCs, DPSCs, and SHED at P5 using Trizol reagent (Invitrogen, Thermo Fisher Scientific, Waltham, MA, USA). The RNA quality was checked by Agilent 2200 (Agilent, CA, USA) and stored at −80 °C. The cDNA libraries were constructed for each RNA sample using the VAHTS Universal V6 RNA-seq Library Prep Kit for Illumina (vazyme, Nanjing, China) according to the manufacturer’s protocol and sequenced by DNBSEQ-T7 on a 150 bp paired-end run. DEGs were analyzed with the following criteria: fold change > 2 or <0.5, *p*-value < 0.05, and false discovery rate < 0.05. GO analysis and KEGG analysis were performed to elucidate the biological implications and significant pathway of the DEGs.

### 4.13. Statistical Analysis

SPSS 20.0 software (SPSS Inc., Chicago, IL, USA) was utilized for statistical analysis. All the statistical calculations were analyzed and expressed as the means and standard deviations. One-way analysis of variance (ANOVA) followed by Tukey’s post hoc test were implemented to identify significant discrepancies among different groups, with statistical significance set at *p* < 0.05.

## 5. Conclusions

This study conducted a comprehensive comparison of three distinct types of DMSCs (SNTSCs, DPSCs, and SHED), focusing on their proliferation, migration, apoptosis, stemness, senescence, and, particularly, odontogenic differentiation potential. Collectively, our findings indicate that both SNTSCs and SHED share many satisfactory characteristics in terms of proliferation and migration. Furthermore, SNTSCs demonstrate superior odontogenic differentiation potential relative to DPSCs but were outperformed by SHED, which was corroborated by functional assays and gene expression analyses. In general, SNTSCs remain a promising cell source besides DPSCs and SHED for endodontic regeneration. Future studies on the immunomodulatory properties of SNTSCs may further optimize the field of application.

## Figures and Tables

**Table 1:** qRT-PCR primer sequence.

	Accession No.	Forward Primer	Reverse Primer
*OCT4*	NM_002701	GTGGAGAGCAACTCCGATG	TGCTCCAGCTTCTCCTTCTC
*SOX2*	NM_003106	GACTTCACATGTCCCAGCACTA	CTCTTTTGCACCCCTCCCATT
*KLF4*	NM_004235	CCATCTTTCTCCACGTTCG	AGTCGCTTCATGTGGGAG
*NANOG*	NM_024865	ATGCCTCACACGGAGACTGT	AGGGCTGTCCTGAATAAGCA
*P16*	NM_000077	CCCCGATTGAAAGAACCAGAGAG	TACGGTAGTGGGGGAAGGCATA
*P21*	NM_001374512	GAGGCCGGGATGAGTTGGGAGGAG	CAGCCGGCGTTTGGAGTGGTAGAA
*P53*	NR_176326	GCCCAACAACACCAGCTCCT	CCTGGGCATCCTTGAGTTCC
*RBL1*	NM_002895	TGGACAGGACTGAACGTCTTG	CCAGCAGGTCAGCAAAGAATTTA
*BCL-2*	NM_000633	GAGGATTGTGGCCTTCTTTG	GCCGGTTCAGGTACTCAGTC
*DSPP*	NM_014208	CAACCATAGAGAAAGCAAACGCG	TTTCTGTTGCCACTGCTGGGAC
*DMP1*	NM_004407	CTGAAGAGAGGACGGGTGATT	CGTGTGGTCACTATTTGCCTG
*ALP*	NM_001632	CCTCCTCGGAAGACACTCTG	GCAGTGAAGGGCTTCTTGTC
*RUNX2*	NM_001024630	CCACTGAACCAAAAAGAAATCCC	GAAAACAACACATAGCCAAACGC
*GAPDH*	NM_002046	GGACACTGAGCAAGAGAGGC	TTATGGGGGTCTGGGATGGA

## Data Availability

The data presented in this study are available on request from the corresponding author, Wei Zhao (zhaowei3@mail.sysu.edu.cn).

## Supplementary Materials

The following supporting information can be downloaded at https://www.mdpi.com/article/10.3390/ijms26051933/s1.

## Abbreviations

SNTSCs	Supernumerary tooth-derived pulp stem cells
MSCs	Mesenchymal stem cells
DPSCs	Dental pulp stem cells
SHED	Stem cells from human exfoliated deciduous teeth
DMSCs	Dental-derived mesenchymal stem cells
CFU	Colony-forming unit
CCK-8	Cell counting kit-8
ALP	Alkaline phosphatase
DSPP	Dentin siolophosphoprotein
DMP-1	Dentin matrix protein 1
RUNX2	Runt-related transcription factor 2
LPS	Lipopolysaccharide
SA-β-gal	Senescence-associated β-galactosidase
OCT4	Octamer-binding transcription factor 4
SOX2	SRY-box transcription factor 2
KLF4	Krüppel-like transcription factor 4
NANOG	Nanog homeobox
RBL1	Retinoblastoma-like 1
BCL-2	B-cell lymphoma-2
PCA	Principal component analyses
DEGs	Differential expression genes
GSEA	Gene set enrichment analysis
GO	Gene ontology
KEGG	Kyoto Encyclopedia of Genes and Genomes
