# Supplementary material for: Molecular Biological Comparison of Pulp Stem Cells from Supernumerary Teeth, Permanent Teeth, and Deciduous Teeth for Endodontic Regeneration
Source: Int J Mol Sci. 2025 Feb 24;26(5):1933. doi: 10.3390/ijms26051933 (PMC11901064; doi:10.3390/ijms26051933)
Supplement: Supplementary file 1 [file ijms-26-01933-s001.zip › ijms-3458221-supplementary.pdf]

Supplementary Materials

Table S1. OD values of SNTSCs, DSPCs, and SHED detected by CCK-8

	SNTSCs	DPSCs	SHED
Day 1	0.2902 ± 0.049907	0.3900 ± 0.073440	0.4058 ± 0.046354
Day 3	0.5008 ± 0.072916	0.5568 ± 0.051066	1.0746 ± 0.141983
Day 5	1.8572 ± 0.159101	1.3992 ± 0.124301	2.9762 ± 0.405001
Day 7	2.0116 ± 0.160539	1.7898 ± 0.136467	3.5198 ± 0.285860

Data are presented as the mean ± standard deviation. n=5

Table S2. Proportion (%) of EdU-positive cells on day 3

SNTSCs	DPSCs	SHED
18.28 ± 5.24	13.04 ± 2.38	24.80 ± 3.03

Data are presented as the mean ± standard deviation. n=5

Table S3. Migrated cells per field in transwell assay

SNTSCs	DPSCs	SHED
65.8 ± 11.52	34.4 ± 4.28	54.8 ± 10.23

Data are presented as the mean ± standard deviation. n=5

Table S4. Quantitative detection of ALP activity (U/grot)

	SNTSCs	DPSCs	SHED
Day 7	135.44 ± 3.04	127.08 ± 1.71	153.83 ± 2.86
Day 14	249.19 ± 37.01	178.17 ± 22.79	282.03 ± 33.35

Data are presented as the mean ± standard deviation. n=3

Table S5. Apoptosis rates after incubation with 10 μg/mL LPS for 24 hours (%)

	SNTSCs	DPSCs	SHED
Q1 (Annexin-V- PI+)	0.18 ± 0.40	0.03 ± 0.15	0.73 ± 1.08
Q2 (Annexin-V+ PI+)	56.6 ± 7.27	30.03 ± 1.30	45.83 ± 4.95
Q3 (Annexin-V+ PI-)	19.09 ± 12.49	17.43 ± 8.33	6.60 ± 4.49
Q4 (Annexin-V- PI-)	24.13 ± 4.90	52.5 ± 7.21	46.83 ± 2.60

Data are presented as the mean ± standard deviation. n=3
